# Ageing in the City: perceptions of urban age-friendliness in three localities of Bogotá, 2023

**DOI:** 10.1186/s12939-026-02808-z

**Published:** 2026-03-11

**Authors:** Yesika Natali Fernández-Ortiz, Rolando Enrique Peñaloza-Quintero, Juliana González-Cortés, Marino Mauricio Mejía-Rocha

**Affiliations:** 1 Pontificia Universidad Javeriana, Institute of Public Health, Bogotá, Cundinamarca 110231 Colombia; 2 Independent Researcher, Bogotá, Colombia

**Keywords:** Ageing, Active ageing, Age-friendly cities, Urban health, Bogotá

## Abstract

Population ageing poses urgent challenges for designing inclusive cities that safeguard the wellbeing of older people. This study analysed perceptions of urban age-friendliness in three localities of Bogotá, guided by the criteria of the World Health Organization’s Vancouver Protocol. An interpretive phenomenological qualitative approach informed the design, using seven focus groups conducted in 2023 with 57 participants (44 older people, 10 caregivers, and 3 service providers). Thematic analysis explored eight dimensions: outdoor spaces and buildings, transportation, housing, social participation, respect and inclusion, employment, communication and information, and social and health services. Findings reveal ambivalent perceptions, with simultaneous experiences of inclusion and exclusion that expose intra-urban territorial inequalities. Participants in Teusaquillo valued available services and support, whereas in Usme and Tunjuelito they identified barriers linked to mobility, insecurity, and deficiencies in infrastructure. Limited access to transport and health services curtailed autonomy, scarce institutional provision reduced opportunities for social participation, and ageism appeared as a cross-cutting theme. Additional factors such as socioeconomic precariousness, the digital divide, and loneliness deepened processes of exclusion. Although Bogotá has advanced towards becoming a more age-friendly city, structural challenges remain that require intersectoral strategies. Strengthening universal accessibility, expanding comprehensive health care, and promoting intergenerational initiatives alongside digital literacy programmes stand out as key actions to reduce social isolation and foster active and equitable ageing.

## Introduction

Population ageing represents a global phenomenon that is reshaping social, economic, and urban dynamics across all regions of the world. This demographic shift generates substantial challenges for designing inclusive urban environments that safeguard the wellbeing and quality of life of older people. In response, the World Health Organization (WHO) developed the Age-Friendly Cities initiative, aimed at promoting active ageing through policies, services, and physical and social environments adapted to the needs of this population. The initiative was later consolidated through the Global Network for Age-friendly Cities and Communities, which encourages the exchange of experiences and lessons learned among cities and communities that have implemented these strategies [1].

According to the WHO, an age-friendly city optimises opportunities for health, participation, and security, thereby improving the quality of life of older people as they age [2]. The conceptual framework of Age-Friendly Cities rests on an ecological perspective of ageing, which links individual wellbeing to the physical and social environment and emphasises enabling older people to age actively and safely within their communities [3, 4].

Within this framework, the paradigm of active ageing has become central to contemporary gerontological, political, and social debates [5, 6]. It is considered one of the main public policy responses to demographic ageing [7]. To guide the implementation of this initiative, the WHO developed the Vancouver Protocol, a participatory tool grounded in the lived experience of older people, which facilitates the identification and assessment of the attributes that define an age-friendly city. The protocol proposes the analysis of eight key dimensions: outdoor spaces and buildings, transportation, housing, social participation, respect and inclusion, employment, communication and information, and social and health services [8, 9].

In 2006, with the purpose of consolidating the initiative, a qualitative study was conducted in 33 cities across 23 countries with diverse urban realities. As a result, in 2007 the WHO published the Global Age-Friendly Cities: A Guide, which, together with the Vancouver Protocol, has served as the methodological foundation for its implementation worldwide [2, 10]. Since then, many cities have adopted this methodology or developed their own approaches to assess and enhance age-friendliness. For example, Bilbao has reported progress alongside challenges in accessibility, employment, and support networks; New York has promoted community engagement through the *Age-Friendly New York City* project; Canada has advanced age-friendliness in rural communities with a dedicated guide; in La Plata, Argentina, cultural and organisational strengths have been highlighted despite ongoing infrastructure and safety problems. In Mexico, assessments in Mexico City and Cancún revealed shortcomings in accessibility and government support, as well as positive aspects in urban planning and mobility. Finally, in Colombia, Ibagué identified persistent difficulties in infrastructure, mobility, safety, and social participation. These experiences reflect the diversity of contexts and needs in building more inclusive urban environments for ageing populations [11].

While these approaches have been instrumental in structuring assessments and guiding policy action, they also raise questions about how age-friendliness is understood and experienced beyond its operational dimensions. Building on this understanding, the present study approaches the Vancouver Protocol as an organising framework rather than a comprehensive explanatory model. The study adopts the notion of ambivalence as an interpretive concept to capture how experiences of inclusion and exclusion may coexist in everyday urban life [12, 13]. Access to services, community ties, or age-friendly programmes may occur alongside insecurity, mobility barriers, institutional discontinuities, or feelings of social invisibility, shaping complex and sometimes contradictory perceptions of ageing in the city.

These ambivalent experiences are not solely the result of individual or local circumstances, but are closely linked to broader structural and policy conditions that shape how age-friendly initiatives are implemented and sustained [14]. In this regard, despite the growth of the Global Network for Age-friendly Cities and Communities, many territories still lack national policies to support urban adaptation to population ageing. Factors such as austerity, inequality, gentrification, and limited political commitment have constrained the effective implementation of this agenda. The COVID-19 pandemic further exposed structural deficiencies in housing, services, and urban environments, highlighting the need for intersectional approaches to address the multiple forms of exclusion faced by older people [15]. This scenario reinforces the urgency of strengthening information, guidance, and regulatory mechanisms that enable cities to implement concrete and sustained actions in this direction.

In large Latin American cities, these challenges unfold within highly heterogeneous urban contexts. Rapid urbanisation has coexisted with persistent social and spatial inequalities, producing uneven living conditions across neighbourhoods and territories [16]. Bogotá is characterised by marked intra-urban inequalities, where access to services, urban infrastructure, and everyday resources varies substantially between localities. In this context, analysing urban age-friendliness requires attention not only to city-level policies, but also to how ageing is experienced locally within the urban territory.

In Colombia, Bogotá ranks among the cities with the highest ageing index. According to the National Administrative Department of Statistics (DANE) [17], in 2021 Bogotá reached an ageing index of 70.2, meaning that there were 70.2 people aged 60 years and over for every 100 people under the age of 15. However, access to services and quality of life for this population remain shaped by deep socioeconomic inequalities [18]. Although academic and institutional efforts have sought to evaluate age-friendliness in Bogotá, gaps persist in understanding how age-friendly initiatives are experienced across different urban territories [19–22]. This study seeks to contribute to this understanding by providing insights derived from an in-depth analysis of three localities with contrasting socioeconomic characteristics.

Within this context, the study selected Usme, Tunjuelito, and Teusaquillo for their socioeconomic diversity and their different levels of population ageing. According to data from DANE (2018) and the Bogotá Health Secretariat (2024) [23, 24] Teusaquillo reports one of the highest ageing indices in the city, while Usme and Tunjuelito concentrate populations in conditions of greater socioeconomic vulnerability and with lower institutional coverage in terms of access to health, social, and municipal services. This territorial diversity allows for the comparison of different realities within the same urban context [25, 26].

From this reality emerges the central research question: How do older people, caregivers, and service providers perceive the age-friendliness of Usme, Tunjuelito, and Teusaquillo in relation to the criteria of the Vancouver Protocol?

To address this question, the study aims to analyse the perceptions of different actors regarding urban age-friendliness in Bogotá, using the eight dimensions of the WHO’s Vancouver Protocol as a reference. Through a qualitative approach based on focus groups, the research seeks to explore how these perceptions, inequalities, and lived experiences shape everyday ageing in the city and inform reflections on active ageing within heterogeneous urban contexts.

In this way, the research not only provides a contextualised understanding of urban age-friendliness in Bogotá but also offers empirical evidence from the city that contributes to the growing body of knowledge on how urban environments can adapt to the needs of ageing populations in Latin America and globally.

## Methodology

### Study design

This study employed a qualitative descriptive–exploratory design with an interpretive phenomenological approach, which enabled an in-depth understanding of how older people, caregivers, and service providers perceive urban age-friendliness in three localities of Bogotá. The research followed the guidelines of the WHO’s Vancouver Protocol and adopted participatory techniques, specifically focus groups structured around this framework, to capture the voices and collective interpretations of the actors involved.

In line with the conceptual approach outlined in the Introduction, the interpretive phenomenological orientation was directed towards how participants made sense of their everyday experiences of ageing within specific urban, social, and institutional contexts. Rather than pursuing a strict phenomenological reduction, the study focused on participants’ interpretations of their interactions with urban spaces, services, and social relations, recognising that these experiences are shaped by broader structural and territorial conditions.

Focus groups served as the primary method because of their suitability for fostering interaction, collective dialogue, and the co-construction of meanings. The study also adhered to the COREQ framework (Consolidated Criteria for Reporting Qualitative Research) [27], which was used as a guide for the reporting and reflexive documentation of the research process. Specifically, COREQ informed the structured presentation of the research team, participant selection, data collection procedures, analytical processes, and ethical considerations, thereby enhancing transparency, reflexivity, and methodological rigour in the reporting of the study. Sessions took place between July and October 2023 in community spaces across the three selected localities, creating a setting of trust and accessibility for participants.

### Research team

The research team consisted of three moderators with backgrounds in public health and social sciences, all with prior experience in qualitative studies with older people. Two women acted as the primary moderators, while one man attended the sessions as an observer to take field notes.

No prior relationship existed between the research team and the participants. Nevertheless, before each session the team presented the study objectives, emphasised their neutral stance, and reaffirmed their commitment to maintaining the confidentiality of the information gathered.

To minimise potential bias in interpretation, the research team held discussion sessions after the initial analysis, where they collectively reflected on the researchers’ roles and on preconceptions that might have influenced data interpretation. They also applied a triangulation strategy among researchers to validate emerging categories and enhance the reliability of the analysis.

The team’s composition—mainly women with extensive experience—may have facilitated trust-building with participants. The researchers acknowledged how their personal and professional trajectories could shape the interpretation of participants’ narratives.

Reflexivity was approached as a transversal and ongoing process throughout the study, acknowledging the interpretative nature of qualitative research and the active role of researchers in knowledge production. Rather than being treated as a separate methodological step, reflexivity informed key decisions during data collection and analysis. During focus groups, attention was paid to the dynamics of interaction and to creating dialogical spaces that allowed participants to articulate their experiences freely, while being attentive to how questions, prompts, and group dynamics could shape the narratives produced. Throughout the analytical process, reflexivity was exercised through iterative engagement with the data, collective discussions within the research team, and the constant questioning of emerging interpretations in relation to the empirical material and the study context. This reflexive practice aimed to make visible the conditions under which meanings were constructed and to enhance the credibility and transparency of the findings, in line with qualitative methodological discussions on reflexivity as a situated and ongoing practice [28].

### Participants

Researchers selected participants through convenience sampling and the snowball strategy, with the support of local community centres that bring together older people, caregivers, and service providers.

Inclusion criteria:

- Older people: aged 60 years or over, residing in one of the three selected localities, and able to participate actively in group discussions.
- Caregivers: responsible for the care of an older person living in one of the localities of interest.
- Service providers: delivering care or services to older people in the selected localities.

Exclusion criteria: individuals with cognitive or health limitations that prevented them from engaging in the focus group discussions did not take part.

Community centres played a key role in facilitating participant recruitment and supporting inclusivity, drawing on staff members’ contextual knowledge of regular attendees. Individuals with cognitive impairments that could hinder participation, as well as those with physical or health conditions that would prevent them from remaining in the sessions for extended periods, were not invited to participate. This process did not involve formal clinical assessments but relied on contextual knowledge to ensure ethical participation and the feasibility of the focus groups.

A total of 57 people participated, distributed into three categories: 44 older people, 10 caregivers, and 3 service providers. None of the sessions recorded refusals or dropouts. Participants in the older people’s category ranged from 60 to 78 years of age, with a gender distribution of 64% women and 36% men. Caregivers were mainly family members, while service providers worked in residential care homes (Table 1).

The study conducted seven focus groups: three (3) with older people, three (3) with caregivers of older people with moderate or severe disabilities, and one (1) with service providers engaged in the care and protection of older people.

Regarding socioeconomic characteristics, older people from Teusaquillo belonged to a middle socioeconomic stratum and mostly had private health insurance and either a pension or financial support from their families. They received assistance with activities such as meal preparation, maintenance of shared spaces, medication management, and mobility support when required.

By contrast, older people who took part in the focus groups in Tunjuelito and Usme lived in dispersed housing—either rented or owned—and belonged to a low socioeconomic stratum. Most were affiliated with the subsidised health scheme, intended for individuals without the capacity to pay and financed through state resources; the remainder accessed the contributory scheme as dependants of a principal contributor, funded through contributions from employees and employers. They did not have pensions, and most continued to work. They carried out daily activities independently and participated in recreational, cultural, religious, and healthcare spaces.

**Table 1:** Demographic Characteristics of Focus Group Participants (*n* = 57)

Characteristics		Older People		Caregivers		Service Providers	
		*N*	%	*N*	%	*N*	%
**Locality**	*Teusaquillo*	16	36%	4	40%	1	33%
	*Tunjuelito*	18	41%	3	30%	1	33%
	*Usme*	10	23%	3	30%	1	33%
**Gender**							
Male		16	36%	4	40%		
Female		28	64%	6	60%	3	100%
**Age (group-years)**							
40–49				2	20%		
50–59				5	50%	3	100%
60–69		27	61%	3	30%		
70–79		17	39%				

Source: Authors’ elaboration

### Data collection

The study drew on the Vancouver Protocol and applied a thematic analysis approach. Before data collection began, participants received an infographic designed to explain the scope of the research and the dimensions of the protocol, which addressed outdoor spaces and buildings, transportation, housing, social participation, respect and inclusion, employment, communication and information, and social and health services. This tool provided a shared conceptual framework for the topics explored during the focus groups.

The focus groups took place in accessible community settings, such as community halls and day centres. During two sessions, participants were accompanied by relatives or caregivers who remained present as support but did not intervene in the discussions.

A semi-structured interview guide, based on the eight dimensions of the Vancouver Protocol, directed the sessions. The guide was piloted prior to its application. All sessions were audio-recorded and initially transcribed using Google Point and NVivo 11^®^, followed by manual adjustments to ensure accuracy. In addition, an observer recorded detailed notes on non-verbal interactions and group dynamics.

Sessions lasted between 60 and 90 min. Thematic saturation occurred when the ongoing analysis of discussions yielded no new categories or substantial variations in the narratives associated with the Vancouver Protocol dimensions. The research team determined this point through a systematic review of the discourses from each actor group (older people, caregivers, and service providers), carried out after each session, by identifying recurring patterns and thematic stability in line with the criteria proposed by Guest et al. (2006) [29].

The interview guide used in this study was adapted from the publicly available WHO Vancouver Protocol, which provides the methodological framework and semi-structured questions for assessing age-friendliness [30].

In keeping with the study’s conceptual positioning, the Protocol was used to structure the topics discussed, while allowing participants to articulate their own experiences, interpretations, and perceived tensions related to urban ageing.

### Data analysis

Thematic analysis was conducted using NVivo 11^®^, following a combined deductive–inductive approach. The initial categories corresponded to the dimensions of the Vancouver Protocol, previously introduced during data collection, which enabled the analysis to be structured around defined conceptual axes. From these dimensions, new subcategories and unanticipated themes emerged, derived from participants’ narratives (Table 2). This interpretive approach sought to capture the meanings that actors attributed to their everyday experiences in the urban environment, in alignment with the study’s phenomenological perspective.

The analysis combined deductive and inductive procedures, allowing predefined dimensions to guide the coding process while remaining attentive to meanings, interpretations, and tensions emerging from participants’ narratives.

In line with the interpretive phenomenological orientation, the analysis extended beyond thematic categorisation to explore how participants described, interpreted, and made sense of their lived experiences of ageing in the city. Particular attention was paid to expressions of ambivalence, understood as the coexistence of inclusion and exclusion, support and constraint, or opportunity and vulnerability within the same accounts. This focus enabled the identification of tensions and contradictions linked to broader social and territorial conditions, which would not be fully captured through a purely descriptive thematic analysis.

Two researchers coded the data independently and then compared their results to ensure coherence and reliability in the process. A codebook guided the analysis, containing the main categories, emerging subcategories, operational definitions, and illustrative examples.

To ensure a contextualised interpretation of the findings, representative verbatim quotations were included to illustrate participants’ experiences and their relation to the analytical categories. In the Results section, the categories of the Vancouver Protocol are developed one by one, with the subcategories and codes narratively integrated, so that the findings emerge in articulation with the participants’ testimonies.

**Table 2:** Subcategories and Codes Grouped by Category

Category	Subcategories	Codes
Outdoor spaces and buildings	- Urban environmental conditions - Pedestrian mobility - Accessible building infrastructure - Safety and urban treatment	- Tranquillity, Odours, Noise, Park maintenance, Access to green spaces, Seating areas - Surfaces, Obstacles, Deteriorated pavements, Unsafe pedestrian crossings and traffic lights -Ramps, Lifts, Stairs - Insecurity in public space, Culture of respect, Treatment in services
Transportation	- Accessibility and cost - Reliability and frequency - Treatment and civic culture in transport - Safety and comfort	- Price, Availability, Non-adapted infrastructure - Service frequency, Reliability, Insufficiency - Disrespect, Lack of courtesy, Inadequate treatment, Civic culture - Overcrowded transport, Alternative transport, Safety
Housing	- Structural and economic conditions - Family strategies and housing options	- Inadequate structure, Adaptations, Comfort, Modifications, Cost of adaptations, Acquisition costs - Capacity, Suitability, Family solutions, Professional support, Family care
Social participation	-Community ties -Participation in decision-making -Limitations and tensions in social inclusion	- Neighbourly respect, Community solidarity - Decision-making, Institutional participation - Verbal violence, Fragmented care, Family support, Public invisibility, Limited public presence
Respect and inclusion	-Range of events and activities -Facilities and environments -Access to information on activities	- Intergenerational activities, Institutional activities - Limited capacity, Conditions of care - Communication problems, Lack of timely dissemination of activities
Employment	-Volunteering opportunities -Employment opportunities -Civic and community participation	- Contribution to society, Absence of programmes, Interest in occupational activities - Informal employment, Age discrimination, Exclusion due to disability - Institutional initiatives, Absence of policies, Lack of state interest
Communication and information	- Information provision - Use and appropriation of technology	- Access to traditional media, Scepticism -Digital technologies, Technological barriers, Family teaching, Learning support, Community information
Social and health services	- Accessibility of services - Service provision, Quality and experience of care	- Difficulty obtaining appointments, Medicine shortages, Distance from services -Imprecise diagnoses, Home health care, Disrespect from health staff, Hospital bureaucracy, Lack of priority treatment.
Emerging categories	- Ageism and discrimination - Emotional wellbeing - Relationship with caregivers - Education and preparation for old age - Socioeconomic precariousness	- Exclusion, Shame in participating, Ambivalent perception - Pets, Loneliness - Empathy, Respect - Tools for old age, Comprehensive training, Digital and health literacy - Structural poverty, Informal work

Source: Authors’ elaboration

### Ethical considerations

The Ethics Committee of the Institute of Public Health at Pontificia Universidad Javeriana approved the research protocol. All participants provided written informed consent. Since the study involved older people, the process included additional measures to ensure comprehension and autonomy: the document was presented in clear and accessible language, read aloud when necessary, and accompanied by opportunities to clarify doubts. The research team confirmed understanding through verbal feedback and emphasised the voluntary nature of participation, including the right to withdraw at any time without consequences.

Data were anonymised to protect participants’ identities. The study complied with Resolution 8430 of 1993 of the Colombian Ministry of Health and adhered to the ethical principles of the Declaration of Helsinki (1964) and its subsequent amendments [31–33].

## Results

Although Bogotá has made progress in building a more age-friendly environment for older people, significant inequalities persist in access to services and living conditions across the city, as illustrated by the experiences reported in the localities of Teusaquillo, Tunjuelito, and Usme. The perceptions of older people, caregivers, and service providers reveal structural and social barriers that restrict active ageing, shaped by a complex interaction of economic, cultural, and urban factors. Verbatim quotations are incorporated throughout the narrative to illustrate these experiences, while Tables 3 and 4 provide a complementary synthesis of subcategories, codes, and representative excerpts.

### Outdoor spaces and buildings

Across the three localities, older people, caregivers, and service providers agreed that the urban environment presents multiple barriers to mobility and the use of public space. Insecurity, deteriorated urban furniture, the lack of accessibility in buildings and pavements, and the poor maintenance of parks and green areas emerged as factors that limit autonomy and participation. Together, these conditions led participants to describe urban space less as a place of integration and more as a restrictive setting for everyday mobility and participation.

In Teusaquillo, participants valued the tranquillity of the environment, which they associated with lower levels of noise, more orderly urban dynamics, and a greater sense of familiarity within the neighbourhood. However, this perception of tranquillity was described as fragile and conditional, as concerns about insecurity remained present: *“The neighbourhood is not safe*,* and it is not safe for older people either”* (woman, 60–69 years, older person, Teusaquillo); *“At this age one has to be very careful”* (man, 70–79 years, older person, Teusaquillo). Thus, tranquillity did not eliminate fear but coexisted with a constant need for vigilance in everyday life. In Usme, participants highlighted environmental discomfort: *“I live in Bellavista*,* and in the neighbourhood there is noise pollution and pollution from odours”* (woman, 60–69 years, older person, Usme). In this contrast, everyday life was described as marked by fears and discomforts that shaped the relationship with the environment.

Green spaces were identified as essential for social interaction, but their poor maintenance and lack of accessibility limited their use. One participant explained: *“As long as there is no constant maintenance of these spaces*,* it is very difficult to use them*,* and worse if the entrances are far away or uneven*,* which makes it hard to get there”* (man, 60–69 years, older person, Teusaquillo).

In addition, participants referred to everyday practical limitations that restricted prolonged stays in these spaces, particularly the scarcity of seating: “*The parks are fine*,* although there are not many benches; the few that exist around here are usable. On the way home there are also a few*,* not many*,* but you do find them”* (woman, 60–69 years, older person, Tunjuelito). The limited availability of benches and resting points was described by participants as a concrete barrier to remaining in public spaces, particularly for those with reduced mobility, underscoring the difficulty of staying in parks in a continuous and safe manner.

Caregivers pointed out that deficient infrastructure particularly compromised pedestrian mobility: *“The Parkway is a very beautiful and pleasant park*,* but the pedestrian path has many holes”* (female caregiver, 50–59 years, Teusaquillo). These limitations were reinforced by short traffic light cycles and unsafe pedestrian crossings, which increased the risk of falls and accidents: *“The traffic lights don’t last long enough for people to cross; it would be better if they lasted longer… The pedestrian crossings are awful*,* full of holes*,* you step and fall…”* (man, 70–79 years, older person, Teusaquillo). As a result, everyday journeys were described less as expressions of autonomy and more as situations requiring constant alertness and self-protection.

Regarding building infrastructure, service providers noted that many urban renovations had failed to incorporate universal accessibility measures such as adequate ramps or lifts: *“Most of the buildings currently being refurbished in the locality do not have lifts*,* and many have staircases without ramps*,* which makes it impossible for people with reduced mobility to enter”* (woman, 50–59 years, service provider, Teusaquillo). Constructions were thus associated more with architectural barriers than with their potential to enable inclusion.

The COVID-19 pandemic further reduced participation in outdoor activities and heightened feelings of vulnerability: “After the pandemic all those kinds of outdoor activities declined”; “They are very vulnerable to anyone approaching them asking for money or robbing them” (woman, 50–59 years, service provider, Usme). More than a temporary interruption, the pandemic left a lasting mark on people’s confidence to take part in activities outside the home.

These accounts indicate that the experience of outdoor spaces was shaped less by their mere presence than by how safety, maintenance, and physical accessibility conditioned older people’s ability to use them autonomously and with confidence.

### Transportation

Older people, caregivers, and service providers described daily experiences of exclusion when using public transport. The main barriers mentioned included the absence of adapted infrastructure in buses and stations, the limited availability of routes, overcrowded vehicles, and the disrespectful behaviour of some drivers. In this context, participants also linked transport difficulties to poor physical conditions along routes, noting that *“the streets are full of holes*,* there are too many works*,* and that makes transport more difficult”* (man, 60–69 years, older person, Usme).

These factors restricted autonomy and led many older people to rely on taxis or digital platforms, although their high cost prevented sustained use. Mobility was narrated more as a source of uncertainty than of trust.

In Teusaquillo, participants criticised the system’s inaccessibility and the inappropriate behaviour of drivers. In Tunjuelito, problems were mainly associated with insecurity, low frequency, and disregard within the Transmilenio system. In Usme, although routes existed, their irregularity and lack of adaptation hindered use, reflecting reliability issues in the service: *“The routes are not sufficient*,* the frequencies are unstable”* (man, 60–69 years, older person, Usme). In many cases, this irregularity forced people to spend several hours on short journeys, increasing dependence on family members or caregivers. For many older people, route instability turned even short trips into long and exhausting journeys. As one participant from Usme explained, “*There aren’t enough buses; you can end up spending three or four hours on journeys”* (woman, 60–69 years, older person, Usme).

The precariousness of the system not only limited mobility but also generated feelings of stress and anxiety. A participant in Tunjuelito summarised it bluntly: “*Getting on public transport is terrible*” (woman, 60–69 years, older person, Tunjuelito). Overcrowded buses and route uncertainty heightened the sense of insecurity and reduced the willingness to move around the city. Congestion generated not only discomfort but also exposure to falls and mistreatment during journeys. Participants described situations in which overcrowding prevented them from boarding vehicles *“When the buses come*,* they are already very full*,* and many of us are left behind because we can’t get on”* (man, 70–79 years, older person, Usme). Even when older people managed to board, journeys were frequently marked by physical crowding and lack of consideration from other passengers: *“On the buses*,* people push you all the time. Once they pushed me far away”* (woman, 70–79 years, older person, Tunjuelito). These situations framed everyday travel as a physically demanding and emotionally tense experience, increasing the perception of vulnerability during mobility.

Beyond these embodied experiences, participants also pointed to broader problems related to treatment and civic culture in transport. Accounts referred not only to pushing, but to the absence of priority and explicit refusals of assistance by drivers, particularly in cases of disability: *“There are drivers who*,* when they see a wheelchair*,* say: ‘I won’t take them*,*’ and they do nothing to help”* (man, 60–69 years, older person, Tunjuelito). Such interactions reinforced feelings of exclusion and illustrated how interpersonal behaviours, together with service responses, contributed to constrained mobility.

As a consequence, many participants reported turning to alternative transport options in search of greater safety and predictability. However, insecurity and high prices also restricted these options. Participants also stressed that route availability was insufficient and unreliable, increasing dependence on private alternatives such as taxis or digital platforms: “*I don’t use public transport; I always take a taxi*,* or my daughter orders me an Uber*,* although it costs me*” (woman, 60–69 years, older person, Teusaquillo). They also highlighted the lack of civic awareness: “*Citizens don’t have the awareness to say*,* I’ll give up my seat*” (female caregiver, 50–59 years, Tunjuelito). Some suggested free fares as an alternative: “*It should be free for older people*” (male caregiver, 60–69 years, Usme). Overall, the accounts portrayed everyday transport as marked by mistreatment, lack of support, and limited adaptation to older people’s needs.

Caregivers confirmed that public transport did not provide adequate conditions and noted that, although taxis were perceived as safer, they were not always financially affordable: “*Here people use taxis a lot; they pick them up at the door and then drop them off here*” (female caregiver, 50–59 years, Teusaquillo); “*My mother doesn’t like travelling on Transmilenio*,* it causes her a lot of stress*” (female caregiver, 50–59 years, Tunjuelito). Thus, while taxis were considered safer, their cost limited frequent use among older people.

Service providers emphasised that insecurity, disrespect, and lack of accessibility had led many older people to avoid public transport altogether: “*Here hardly anyone goes out to take buses or Transmilenio anymore*” (woman, 50–59 years, service provider, Teusaquillo). They also underscored the structural deficiencies of buses: “*The buses don’t have ramps… and when they do*,* the drivers see them and keep going*” (woman, 50–59 years, service provider, Tunjuelito). According to service providers, current transport conditions make it an unreliable service, disconnected from the needs of older people.

Transportation emerged as a key mediator of independence, where affordability, reliability, and interpersonal treatment intersected to either enable or restrict participation in everyday urban life.

### Housing

Homes did not always provide safe or accessible conditions for ageing with dignity. Older people described physical obstacles that increased the risk of falls, as well as economic constraints that made necessary adaptations difficult. In many cases, modifications depended on family creativity or informal networks that sought to compensate for the absence of institutional support. Rather than functioning solely as spaces of protection, homes were often described by participants as reflecting economic precariousness and limited institutional support, particularly through the absence of publicly supported housing adaptations and sustained professional or institutional assistance.

The main deficiencies identified included the absence of handrails, non-slip flooring, and adequate lighting—elements that increased the risk of falls and accidents. These limitations were compounded by the high cost of materials and labour, which reduced the feasibility of adaptations: *“The materials are very expensive*,* and so is the labour”* (man, 60–69 years, older person, Usme). In some cases, structural constraints further limited possibilities for change: “*I’d like to fix my room*,* but I can’t because of a column*,* and the cost is very high—we don’t have enough money*” (woman, 60–69 years, older person, Tunjuelito).

For those who were not homeowners, access to adequate housing was even more limited: *“For those of us who don’t have a house*,* it is very difficult to get one”* (man, 60–69 years, older person, Usme). Homes were thus described as difficult to adapt and as spaces that sustained persistent barriers in daily life.

Despite these constraints, perceptions of comfort were not absent. In Teusaquillo, some participants described feeling at ease in their living environments: *“I live in a four-storey building; it is very peaceful and comfortable where I live. I don’t know what it’s like in other places”* (woman, 70–79 years, older person, Teusaquillo). However, such perceptions of comfort often coexisted with domestic settings that were not fully adapted or safe, revealing a tension between subjective wellbeing and objective housing conditions.

In Teusaquillo, service providers reported having more adapted infrastructure, including lifts and ramps, as well as professional support that reinforced the capacity and relevance of services: *“Modifications were made*,* and we have a lift”* (woman, 50–59 years, service provider, Teusaquillo). They also highlighted professional support as a protective factor: *“The support they provide here is spectacular”* (woman, 50–59 years, service provider, Tunjuelito). These accounts illustrate institutional support in concrete terms, including publicly or organisationally supported housing adaptations and the availability of professional assistance.

In Tunjuelito and Usme, however, although many older people reported feeling comfortable in their homes, these did not always provide safe conditions. Caregivers explained: *“The floors are very rustic and not suitable”* (female caregiver, 50–59 years, Tunjuelito); *“People live in inadequate houses”* (female caregiver, 40–59 years, Usme). Perceptions of comfort thus coexisted with domestic settings that were unsafe or poorly adapted.

When home modifications were not possible, housing-related constraints were often addressed through domestic and family-based solutions, reflecting strategies of resistance within the household: *“The way we found for my mother to call for help was to buy her an emergency bell”* (female caregiver, 50–59 years, Tunjuelito). In this context, family support emerged as the most stable resource to guarantee daily companionship and safety within the home, and in some cases involved shared housing arrangements: *“I live with my daughter*,* so she looks after me and I don’t have to pay rent. Here I am close to my grandchildren and I feel accompanied”* (woman, 70–79 years, older person, Usme). However, these family strategies were described as palliative and insufficient in the absence of publicly supported housing adaptations and sustained professional assistance.

### Social participation

Social participation is understood as the everyday practices through which older people engage with community life, including neighbourhood relations, family interactions, and involvement in local activities.

Although social spaces that encourage interaction exist in all three localities, older people continue to face barriers that hinder their real inclusion. The narratives revealed tensions between the desire to participate, and the limitations imposed by lack of recognition, family fragmentation, and limited visibility in community life. Participation was perceived as irregular and dependent on isolated local efforts.

In Usme and Tunjuelito, Community Action Boards (Juntas de Acción Comunal, JAC) and local administrations organised community activities that promoted social integration, although these were not always specifically targeted at older people, reflecting restricted access to institutional participation. In Teusaquillo, however, some accounts pointed to more active forms of involvement, with service providers noting that *“some community councils*,* boards*,* and associations are run by older adults here in the locality”* (woman, 50–59 years, service provider, Teusaquillo). Nevertheless, participants also noted tensions in intergenerational spaces, and in many cases activities were experienced as one-off opportunities without sustained continuity.

Perceptions of community participation showed nuanced variations across localities, particularly in how neighbourly support was experienced and valued. In Usme and Tunjuelito, relationships of respect and everyday closeness were highlighted as a source of mutual support: “*I have always got on well with all my neighbours; they are very good people*” (woman, 60–69 years, older person, Tunjuelito). In Teusaquillo, participation was more often framed in terms of attentiveness and practical assistance within the immediate environment: “*I feel good because my neighbours are attentive to my needs*” (man, 70–79 years, older person, Teusaquillo). Across these contexts, neighbours’ willingness to help when needed reinforced a shared sense of community closeness, underscoring the role of neighbourhood networks as a key form of everyday support, albeit expressed through locally specific practices and expectations.

Within the family sphere, inclusion in decision-making was recognised as significant for wellbeing, as it conveyed a sense of recognition, agency, and continued social relevance in everyday life. Being consulted on domestic matters reinforced the feeling of still being able to contribute meaningfully: *“My family takes me into account when making decisions*,* especially my children”* (woman, 60–69 years, older person, Teusaquillo).

Similar experiences of recognition were also described in community-based settings, particularly in organised activities where older people felt listened to and actively involved: *“They take us into account. Sometimes they ask us what we need*,* and they come to teach us things*,* and that makes us feel that we really can participate”* (woman, 60–69 years, older person, Tunjuelito). In both contexts, participation was associated less with formal roles and more with the experience of being acknowledged as a legitimate voice.

However, alongside these experiences of recognition, participants also described forms of social discrimination that undermined their sense of belonging and willingness to participate in everyday social life, including episodes occurring in public settings that function as key spaces of social interaction. One participant recounted an episode of public humiliation: *“When I got on the bus and went to take a seat*,* some young people pushed me to the floor and said: you look like a cow”* (woman, 70–79 years, older person, Usme). Beyond such overt incidents, other accounts referred to more subtle but recurrent forms of verbal ageism embedded in everyday language, such as being addressed as “Cucho” or “Cucha,” expressions that participants associated with disrespect and social devaluation.

These experiences were narrated by participants not merely as isolated acts of aggression, but as part of a broader repertoire of everyday interactions that reinforced stigmatisation and constrained older people’s participation in public and community life.

From the perspective of service providers, although ageing was valued within the community, their accounts highlighted perceived challenges in coordinating with other institutional actors, which affected the availability of adequate spaces and sustained opportunities for participation: “*We need more unity*,* more dialogue*,* more saying let’s not do this*,* let’s do that*” (service provider, Usme). They also described family practices that could undermine emotional stability, such as rotating older relatives between households: “*The children sell the house and the mother is rotated among them*” (service provider, Teusaquillo). Other accounts referred to more distant care dynamics, where family visits were limited to sporadic contact. Nonetheless, examples of family commitment and accompaniment also emerged: “*Each week one of her sons comes to accompany her*” (female caregiver, 50–59 years, Tunjuelito). Family relationships thus oscillated between gestures of support and situations of neglect.

Caregivers stressed that society still affords little recognition to older people: “*There needs to be more awareness*,* that as a society we give more importance to older people*” (male caregiver, 60–69 years, Tunjuelito). Although JACs organised community events, these did not always effectively include them: “*The president of the Board organises activities for children or for Mother’s Day*,* but older people are not really taken into account*” (female caregiver, 50–59 years, Usme). This lack of consideration in collective spaces reinforced feelings of public invisibility.

These narratives suggest that social participation was experienced as uneven and fragile, sustained primarily through informal community ties rather than through stable or continuous institutional support.

### Respect and social inclusion

While participation refers to involvement in everyday social practices, respect and social inclusion relate to the conditions (symbolic, institutional, and infrastructural) that enable or constrain such participation.

Older people’s experiences reflected fragmented inclusion. Although some initiatives fostered community cohesion, the availability, accessibility, and continuity of activities varied significantly across localities and did not always respond to the real capacities of those involved. Overall, these initiatives were described in participants’ and caregivers’ accounts as temporary and inconsistent efforts.

In Usme, activities organised by the Community Action Boards strengthened social integration: “*I feel well treated in the neighbourhood; the president of the Board takes us into account*” (man, 60–69 years, older person, Usme). In Tunjuelito, recreational events were also valued for their impact on wellbeing: “*I am doing exercises with other older people*,* and they are excellent*” (woman, 60–69 years, older person, Tunjuelito). Community activities were narrated as spaces that foster closeness and contribute to wellbeing.

In Teusaquillo, perceptions of intergenerational activities were ambivalent: while some enjoyed interaction with children and young people, others found it problematic because of noise and lack of organisation: “*I like the activities where they bring children and young people*” (woman, 60–69 years, older person, Teusaquillo) / “*It is very annoying because they make a lot of noise and disorder*” (woman, 70–79 years, older person, Teusaquillo). Some participants also noted that although certain institutional contexts created spaces for listening, in other environments—such as religious ones—there were no specific programmes for older people. The accounts showed that intergenerational coexistence combined moments of satisfaction with everyday tensions.

Service providers promoted cognitive stimulation and recreational programmes, although their continuity depended on resources and political will: “*It is about providing activities so that they keep their minds occupied as much time as possible*” (woman, 50–59 years, service provider, Teusaquillo). They also highlighted communication issues, since lack of information limited attendance: “*There needs to be more information about what is going to be done and the schedule*” (woman, 50–59 years, service provider, Usme). Experiences revealed that these activities tended to fade away due to lack of resources and continuity.

Caregivers emphasised infrastructural limitations, pointing out that spaces were insufficient for the demand: “*I submitted the paperwork six months ago*,* and they haven’t called me because the place is too small*” (female caregiver, 50–59 years, Usme). They also observed that the conditions of care did not always adjust to the capacities of older people: “*They didn’t have patience when she could no longer touch her knees*,* and they gave her no other instruction*” (female caregiver, 50–59 years, Tunjuelito). These difficulties worsened in cases of disability, where the lack of equipment and staff further restricted inclusion. In several accounts, the available spaces were described as insufficient and poorly adapted to the capacities of older people, not merely as physical limitations but as conditions that effectively restricted inclusion and participation.

In this sense, respect and social inclusion were not described as abstract values, but as everyday conditions reflected in interpersonal interactions, access to information, and the perceived recognition of older people in public and institutional spaces.

### Employment and volunteering

In all three localities, older people expressed a recurring desire to remain active, either through paid work or volunteering. This participation was associated not only with economic needs but also with the wish to feel useful and recognised. However, they faced structural and social barriers that limited their inclusion, such as the absence of specific programmes, age discrimination, and labour informality. In their accounts, work and volunteering appeared more as aspirations than as actual opportunities.

Volunteering was perceived as a way of contributing to society without financial remuneration, generating emotional and social benefits. As one participant explained: *“It is a job that a person does willingly because they want to*,* and sometimes it has no economic remuneration”* (man, 60–69 years, older person, Teusaquillo). Some older people expressed interest in supporting vulnerable groups, although their own health conditions limited that possibility: *“I would like to accompany a child*,* but I hit my head… now I am not capable”* (woman, 70–79 years, older person, Teusaquillo). Despite this interest, participants highlighted the absence of structured programmes in Bogotá: *“In Colombia there are no volunteering programmes; in the United States there are”* (man, 60–69 years, older person, Teusaquillo). They also recognised experiences linked to community and cultural activities, valued for their social impact. Narratives described volunteering as an occasional practice, with few formal opportunities available in the city.

Regarding employment, many older people wished to continue working, either out of economic necessity or to remain occupied, but faced hiring barriers: *“I would like to work with the sewing machine*,* but nobody hires you”* (woman, 60–69 years, older person, Teusaquillo). In response, they turned to informal jobs without social guarantees: *“I work in a small shop*,* but it is not legal employment*,* it’s because of my age”* (man, 60–69 years, older person, Usme). Those with disabilities encountered even greater obstacles, opting for self-employment: *“Once you have a disability*,* there is no work; you have to set up your own business”* (man, 60–69 years, older person, Usme). In most cases, income in old age came from informal or subsistence work.

Service providers agreed that volunteering depended on community or religious initiatives, whose continuity was limited by the lack of institutional support. They recalled past experiences such as the Time Bank: “*Years ago*,* there was something called the Time Bank*,* where you could donate your time in nurseries*,* hospitals*,* or with older people. I knew cases of people who recovered emotionally thanks to it. Today it no longer exists*,* but it was a very valuable initiative*.” (woman, 50–59 years, service provider, Teusaquillo) At the same time, they acknowledged older people’s interest in staying active: “*Here everyone wants to keep busy doing things: one knits*,* another paints*” (woman, 50–59 years, service provider, Teusaquillo). Past initiatives were remembered as valuable opportunities that are no longer available today.

Caregivers stressed that although older people showed interest in participating, no public policies promoted their integration into work or volunteering: “*There is not enough for them to be volunteers*,* but they can be included everywhere*” (female caregivers, 50–59 years, Usme). Many resorted to informal survival strategies: “*Many older people go out to sell small bags to raise money for rent*” (female caregivers, 50–59 years, Tunjuelito). This situation reinforced perceptions of state neglect among caregivers and older participants, as reflected in their accounts of the absence of employment or volunteering policies aimed at older people: “*The State has never created an effective plan for older people to have employment; it should be paid*” (female caregivers, 50–59 years, Tunjuelito). The economic activities available were described as unstable and lacking social protection. Caregivers noted that the work opportunities accessible to older people were usually informal and without guarantees.

**Table 3:** Subcategories, Codes, and Verbatims from the Categories Outdoor Spaces, Respect and Inclusion, and Employment Categories

Subcategory	Code	Representative Verbatim
***Outdoor Spaces and Buildings***		
Accessible building infrastructure	Lifts	“Sometimes it is a struggle to get the lift, you have to queue for a long time and you always end up waiting.” (woman, 70–79 years, older person, Usme)
Safety and urban treatment	Culture of respect	“Going out on the street to exercise is almost impossible because older people are not respected. Car and motorcycle drivers don’t take precautions: they scare them, pass too close, or almost run them over. They don’t respect the roads, and this makes insecurity terrible for them.” (woman, 50–59 years, service provider, Tunjuelito)
***Respect and Social Inclusion***		
Range of events and activities	Institutional activities	“My mother goes to church; she is not really someone who participates much, and there are no activities for older people there either.” (male caregiver, 50–59 years, Tunjuelito)
Facilities and environments	Limited capacity	“The day centre is very small, and although they hold activities on the steps, there are too few places for the number of people.” (woman, 60–69 years, older person, Tunjuelito)
	Conditions of care	*“*My father was not admitted because he uses a wheelchair. They told me the machines were broken and there was nobody to operate them. They did not offer another option for him to be with other people.*”* (female caregiver, 50–59 years, Tunjuelito)
Access to information on activities	Communication problems	“At the community hall there are workshops, but when you go to ask, they always say there are no places. You hand in the paperwork, and then they don’t call you. Sometimes you even have to take the photocopy again, because they are so disorganised with that.” (man, 60–69 years, older person, Usme)
***Employment and Volunteering***		
Volunteering options	Contribution to society	“A lady who came as a volunteer… she would come and sing to them.” (woman, 50–59 years, service provider, Teusaquillo)
Employment options	Age discrimination	“They don’t hire you even to peel a potato. They say you are too sweet, that you’re no good anymore, and they scold you for not doing it right. With so many young people looking for work, they reject you because of your age.” (man, 60–69 years, older person, Usme) “Getting a job now is very difficult. Older people are pushed aside. Before there were more opportunities, but nowadays, if young people can’t find work, much less can we.” (woman, 70–79 years, older person, Tunjuelito)
	Informal employment	“I didn’t manage to get a pension; I work by the day. The important thing is to have money to pay expenses.” (man, 60–69 years, older person, Usme)

Source: Authors’ elaboration

### Communication and information access

Access to information among older people was shaped by both generational and technological divides. While many continued to rely on traditional media such as radio and television, others had begun to explore digital tools with the support of family members or institutions. However, this transition was experienced unequally, with obstacles ranging from distrust of content to technical difficulties in using devices.

Traditional media remained the main source of information, due to the trust and familiarity they inspired: *“You have to stay tuned to the radio and television so that information reaches you”* (woman, 70–79 years, older person, Teusaquillo). In some cases, family members complemented what was heard on the radio or television. Although some participants valued the mobile phone: *“I like the mobile phone because I can listen to music*,* watch the news*,* and talk to other people”* (woman, 60–69 years, older person, Tunjuelito), others acknowledged limitations: *“I don’t know how to use it as well as other people”* (man, 60–69 years, older person, Usme). Thus, the use of digital tools coexisted with a strong reliance on traditional channels.

Distrust of digital media was also recurrent. Several participants expressed concerns about violence or media manipulation: *“The news makes you very afraid*,* massacres*,* robberies*,* assaults”* (Tunjuelito); *“There’s nothing reliable*,* because the news sometimes says whatever it wants”* (Usme); “I hardly watch television anymore. I feel they have confused my head with so much, and I prefer not to know anything.” (Tunjuelito). This scepticism translated into reduced consumption of digital or televised information.

This ambivalence coexisted with adaptation processes, usually facilitated by family members or institutions: *“My son taught me that you can ask the mobile phone anything”* (man, 70–79 years, older person, Teusaquillo). Some service providers implemented basic training: *“They gave them computing classes*,* and afterwards it was easy for them to use the computer”* (woman, 50–59 years, service provider, Teusaquillo). In care homes, WhatsApp groups expanded the diffusion of events: *“Now the internet is being used a lot*,* through WhatsApp chains”* (woman, 50–59 years, service provider, Teusaquillo). Participants also noted that some institutional training opportunities had disappeared, reducing chances for digital learning. Digital learning was possible mainly through family or community support, while local networks continued to complement access to reliable information.

From caregivers’ perspectives, many older people preferred to continue using traditional media because of their ease of use: *“They don’t know how to use technology; older people can’t manage a mobile phone”* (female caregiver, 50–59 years Tunjuelito). Even those who had learned sometimes struggled to control what they received: *“My mother already knows how it works*,* but she downloads things without knowing where they come from”* (female caregiver, 40–49 years, Usme). These barriers became more pronounced when devices did not match prior experiences, generating frustration rather than inclusion.

Oral communication remained an essential channel of access to information: *“When he goes out*,* his friends tell him what has happened”* (male caregiver, 60–69 years, Tunjuelito). Interaction with neighbours, friends, or community leaders complemented access to news and experiences, reinforcing trust in immediate sources. These accounts showed that, beyond the digital sphere, community life continued to be a central axis for staying informed.

### Social and health services

Conditions of access to healthcare reflected profound territorial inequalities. While some older people reported experiences of close and responsive care, others faced multiple obstacles such as delays in appointments, shortages of medicines, and institutional mistreatment. Participants’ narratives portrayed a fragmented experience of care, marked by perceived discontinuity and limited coordination across services.

In Teusaquillo, proximity to health centres and the possibility of using private services represented an advantage for some, although the perceived quality depended on the affiliation regime. Despite this relative advantage, access to specialised care remained problematic: *“The problem is that at the EPS there are never appointments available. I needed an appointment with neurology and have never been able to get one”* (woman, 60–69 years, older person, Teusaquillo). In Tunjuelito and Usme, by contrast, participants reported greater difficulties in obtaining medical appointments: “*I have been asking for an appointment for almost a year*,* and they always tell me it is on a waiting list*” (man, 70–79 years, older person, Tunjuelito). Accounts from participants in the three localities pointed to similar difficulties in accessing specialised services, where obtaining appointments was described as particularly challenging. In several cases, participants reported having to resort to legal action (tutelas) in order to access specialist care.

Medicine delivery was also highlighted as a barrier: “*I go to collect them*,* and they say: these medicines are available*,* and these are not*,* until they expire*” (woman, 70–79 years, older person, Tunjuelito). These problems were compounded by staff shortages and the absence of priority care, which forced older people to wait for long hours in queues to obtain their treatments: “*Booking appointments is difficult*,* and the queues to collect medicines are dreadful*” (man, 60–69 years, older person, Usme). In many cases, families had to cover additional costs, allocating part of pensions to buy medicines. Shortages and long queues were described as obstacles that interrupted the continuity of treatments.

Although hospital care could be adequate, several participants described recurrent experiences of initial misdiagnosis or delayed diagnosis, particularly in first levels of care, which contributed to perceptions of fragmented and discontinuous care: “*I went to the doctor*,* they gave me medication*,* but I didn’t get better. Then I went to the emergency department*,* and there they diagnosed me correctly*” (man, 60–69 years, older person, Usme). From the perspective of service providers, some care homes had established agreements with insurers (EPS) to facilitate home-based care: “*Here we work with many EPS and*,* in general*,* the care is good*” (woman, 50–59 years, service provider, Teusaquillo). Nevertheless, several caregivers noted the disappearance of home follow-up programmes, which reduced the possibility of continuous care. Narratives combined accounts of delayed diagnoses with positive perceptions of some home-based services.

However, care in external clinics continued to be slow, reflecting hospital bureaucracy: “*When they arrive at the hospital or clinic*,* it is another matter—the care is delayed*” (woman, 50–59 years, service provider, Teusaquillo). This was compounded by disrespectful attitudes from healthcare staff: “*I had to file a complaint because of the mistreatment of the grandfather*” (woman, 50–59 years, service provider, Teusaquillo). Healthcare was described by participants and caregivers in terms of slowness, bureaucracy, and lack of respect.

Caregivers emphasised that distance to health centres constituted a structural difficulty: “*They attend to him in the north*,* and we live in the south*” (male caregiver, 60–69 years, Tunjuelito). Even where infrastructure existed, it was not always functional: “*The facility has a ramp*,* but it is very steep*,* and I need two people to help push the wheelchair up*” (female caregiver, 50–59 years, Usme). Family caregivers described how distance and physical barriers further complicated access to healthcare.

Many also reported the absence of priority queues for medicine collection: “*They have no consideration for them; there is no priority line*” (male caregiver, 40–49 years, Tunjuelito). Faced with these barriers, some caregivers argued for strengthening home-based care: “*The health system should provide follow-ups at home and have professionals who visit people who can no longer go to the doctor*” (female caregiver, 40–49 years, Tunjuelito). Home care was narrated as a useful resource, although insufficient to meet all care needs.

Collectively, these experiences portray access to social and health services as a source of both support and frustration, shaped by procedural barriers and interpersonal encounters rather than by service availability alone.

### Cross-cutting experiences shaping urban age-friendliness

Beyond the dimensions established by the Vancouver Protocol, the analysis identified a set of cross-cutting experiences that permeated multiple domains of urban life rather than constituting an additional dimension of age-friendliness. These experiences emerged across participants’ narratives and help contextualise how age-friendliness was lived and interpreted in everyday settings.

Themes such as age discrimination, socioeconomic precariousness, emotional wellbeing, and the need for preparation for later life cut across participation, inclusion, and access to services. Participants described experiences of symbolic ageism and social invisibility alongside material forms of exclusion linked to precarious living conditions, informal work, or begging. Emotional wellbeing was frequently associated with relational dimensions, including companionship with pets or experiences of loneliness, highlighting how affective and symbolic factors shaped ageing in the urban context.

These cross-cutting experiences were recurrently mentioned by participants and contributed to ambivalent perceptions of age-friendliness across the three localities. While some participants valued neighbourhood solidarity and everyday gestures of care— “*I feel good because people are kind and good-hearted*” (woman, 60–69 years, older person, Teusaquillo) — others emphasised persistent barriers related to insecurity, lack of respect, and inadequate infrastructure: “*They don’t respect a person with a disability*” (man, 70–79 years, older person, Tunjuelito). Quality of life thus appeared as a fragile balance between community support and structural constraints.

Living conditions and available resources further shaped these experiences. For some, pets provided emotional support and companionship: *“I have my little dog who accompanies me everywhere and makes me happy”* (woman, 70–79 years, older person, Teusaquillo) —while others highlighted situations of extreme vulnerability associated with the absence of housing, income, or social protection: *“There are many older adults on the streets begging*,* without healthcare or protection”* (woman, 50–59 years, service provider, Tunjuelito). Loneliness emerged both through family fragmentation and through the search for affective bonds in non-human companionship.

Age discrimination was often described in subtle and normalised forms, such as exclusion from services or lack of consideration in public spaces: *“They don’t discriminate openly*,* but not providing care is already discrimination”* (female caregiver, 50–59 years, Tunjuelito).

From the perspective of service providers, the importance of strengthening the caregiver–older person relationship was emphasised, grounded in vocation and commitment: *“One must have vocation*,* willingness*,* commitment*,* and responsibility”* (woman, 50–59 years, service provider, Tunjuelito). Participants also stressed the need for education and preparation for later life, including learning opportunities related to health, digital skills, emotional regulation, and social participation: *“Education is needed on health*,* finances*,* emotions*,* and relationships”* (woman, 60–69 years, older person, Teusaquillo). Some participants pointed to preparation for old age as a collective process of care and learning over time.

Taken together, these cross-cutting experiences illustrate how material conditions and symbolic meanings intersected to shape the experience of ageing in the city. Rather than functioning as isolated issues, they operated across domains of urban life, helping to explain the ambivalent and uneven perceptions of age-friendliness observed across the three localities.

**Table 4:** Subcategories, Codes, and Verbatims in Communication, Information, Social and Health Services, and Emerging Categories

Subcategory	Code	Representative Verbatim
**Communication and Information**		
Use and appropriation of technology	Technological barriers	“They are used to their basic mobile phone, and then on their birthday or Mother’s Day, they get given a smartphone, which only complicates their lives because the smartphone is far too difficult for them.” ((woman, 50–59 years, service provider, Teusaquillo)
	Community information	“People from the church and the pastor tell you the news.” (woman, 70–79 years, older person, Tunjuelito) “You find out what is happening from what neighbours or family say. That’s how you stay informed about the situation in the neighbourhood.” (man, 60–69 years, older person, Usme)
	Support for learning	“There used to be a municipal place where they ran educational activities for older people, but now they turned it into a health centre, and they don’t offer anything for them anymore.” (female caregiver, 50–59 years, Teusaquillo)
**Social and Health Services**		
Access to services	Distance from services	“Going to those medical service places is not easy. You always have to spend twice the time to get there.” (woman, 60–69 years, older person, Usme)
Service provision, quality, and experience of care	Disrespect from health staff	“Some doctors are very rude. They ask you reluctantly: ‘What hurts, grandad? What do you need?’ and they don’t even examine you. Not all are like that, but most are.” (man, 70–79 years, older person, Tunjuelito)
	Lack of priority care	“There should be a special line for us, where with our medical history they already know what we need and prepare the medicines. They should prioritise appointments more and not make us suffer so much.” (woman, 60–69 years, older person, Usme)
	Home-based healthcare	“The government should have staff who go around the neighbourhoods visiting people who can no longer go to the doctor.” (man, 70–79 years, older person, Usme)
**Cross-cutting experiences shaping urban age-friendliness**		
Ageism and discrimination	Exclusion	“They don’t respect a person with a disability. If you can’t walk fast, here they just push past you.” (man, 70–79 years, older person, Tunjuelito) “Nowadays they love a dog more than an old person. Sometimes you feel forgotten, but animals are given everything.” (woman, 60–69 years, older person, Usme)
	Shame in participating	“I don’t go to those classes because I feel embarrassed. Everyone else is younger or in better condition, and sometimes they even look at you strangely when you don’t understand quickly.” (woman, 70–79 years, older person, Tunjuelito)
Emotional wellbeing	Loneliness	“I get up and the first thing I do is talk to the cat. At least it listens to me. In this house, it is the only one that is always here.” (woman, 70–79 years, older person, Usme)
Relationship with caregivers	Empathy and respect	“Caring for older people can be pleasant, even fun. Each one has their quirks, and for the work to flow, you must do it with love, vocation, willingness, commitment, and responsibility.” (female caregiver, 50–59 years, Teusaquillo)
Education for ageing	Digital and health literacy	“It would be good if they taught us technological things with young people, so we could learn from each other. We can also contribute, but sometimes you feel left aside.” (woman, 60–69 years, older person, Teusaquillo)
Socioeconomic precariousness	Structural poverty	“Nowadays, older people are left out on the streets picking from the rubbish because they have no way to eat.” (woman, 50–59 years, service provider, Tunjuelito)

Source: Authors’ elaboration

## Discussion

The findings discussed in this section point to an ambivalent experience of urban age-friendliness in Bogotá. Across the three localities analysed, participants described everyday lives shaped by simultaneous processes of inclusion and exclusion, suggesting that age-friendliness remains a condition under construction rather than a consolidated urban reality. Differences between Teusaquillo, Usme, and Tunjuelito were central to these perceptions, as access to services, infrastructure, and opportunities for participation varied markedly across territories, shaping autonomy and quality of life in uneven ways.

In relation to outdoor spaces and housing, participants expressed contrasting perceptions. In Teusaquillo, the relative tranquillity of the environment and neighbourhood networks were valued, although these coexisted with ongoing concerns about insecurity. In contrast, in Usme and Tunjuelito, concerns prevailed around insecurity, deteriorated urban furniture, and inadequate housing. The literature has highlighted the importance of the physical environment for wellbeing in later life [34, 35]. However, in vulnerable contexts participants’ accounts suggest that these barriers extend beyond physical conditions and are compounded by limited economic resources to make adaptations, forcing families into improvised strategies. In this regard, experiences of community-based tactical urbanism and greening projects have demonstrated positive effects on neighbourhood revitalisation and safety [36–40].

Mobility and transport constituted another axis of exclusion. Although limited accessibility and congestion were reported in all localities, the obstacles were more severe in Usme and Tunjuelito, reflecting clear territorial inequity. Unlike some international studies that report advances in inclusive transport design within specific urban contexts [41–43], in Bogotá mobility was described as stressful and reliant on costly alternatives such as taxis or digital platforms. Beyond technical aspects, narratives revealed that mobility is also a cultural issue, conditioned by civic practices that reinforce exclusion, such as refusing to give up seats or treating older passengers with hostility. In light of participants’ accounts describing mobility as stressful, exclusionary, and reliant on costly alternatives, the literature proposes measures such as preferential buses and routes for older people, as well as public awareness campaigns [44, 45].

Social participation and respect/inclusion emerged as strongly ambivalent domains. Some older people highlighted positive neighbourhood and family ties, while others experienced institutional invisibility, verbal violence, and shame. This tension adds to the regional literature by showing that social imaginaries and symbolic barriers can be as limiting as the lack of infrastructure [46, 47]. Recreational or intergenerational activities exist, but they were described in participants’ accounts as fragmented, discontinuous, and not always adapted to their capacities, reinforcing perceptions of exclusion. Experiences in New York, Japan, and Italy demonstrate that sustained intergenerational programmes strengthen social cohesion and improve perceptions of ageing [48–51].

Employment and volunteering were also experienced unequally. In Teusaquillo, some older people accessed occasional volunteering opportunities, while in Usme and Tunjuelito most resorted to informality or self-employment out of economic necessity. International literature recognises the value of volunteering for wellbeing [52–54]. In Bogotá, however, such opportunities remain marginal, and participants interpreted this marginality as being related to limited policy support for older people’s participation in labour and community life. Highlighted solutions in other contexts include flexible employment programmes and intergenerational mentoring schemes [55, 56].

In communication and information, narratives highlighted the existence of a “grey digital divide,” understood as the combination of technological, trust-related, and usability barriers that limit older people’s inclusion in community life [57]. Unequal access to information deepens inequalities and restricts rights, while digital inclusion—today recognised as a social determinant of health—shapes the capacity to access services and participate in community life [58, 59]. Although some older people used mobile phones and social media, difficulties in handling technology, distrust of digital media, and preference for traditional channels persisted, making this divide a factor of exclusion. Previous studies have highlighted digital literacy as a pathway to social inclusion [60]. This study contributes by showing that community networks and oral communication remain central, underscoring the need for intergenerational digital literacy programmes tailored to older people [61–63].

Access to social and health services was one of the most critical and unequal challenges. In Teusaquillo, some participants accessed private or nearby services, while in Usme and Tunjuelito they reported long waits, medicine shortages, and lack of sensitivity from staff, which affected continuity and quality of care. Moreover, caregivers faced emotional exhaustion and financial strain. These differences reinforced participants’ perceptions of health inequities as intertwined with both limitations in service provision and the uneven urban distribution of resources [64, 65]. International experiences support the effectiveness of integrated primary and home-based care models, which have reduced hospital pressure and improved equity in access [66–68].

Taken together, the findings suggest that two interconnected axes organised participants’ accounts across domains: intra-urban territorial inequality and ageism. Rather than operating independently, these axes cut across the dimensions explored and help explain why urban age-friendliness was experienced as uneven and, at times, contradictory within the same city.

Within this framework, ageism emerged as a particularly relevant transversal process across domains. Although initially identified as a subcategory in the analysis, participants’ narratives indicate that ageism operates as a structuring condition of everyday urban life, shaping how older people move, participate, access services, and are recognised in public and institutional spaces. Accounts referred to lack of consideration in urban environments, mistreatment in transport, derogatory attitudes in healthcare, exclusion from employment opportunities, and the reinforcement of digital exclusion through assumptions about older people’s inability to learn.

These findings are consistent with international research showing the impact of ageism on social integration [69–71]. However, this study adds nuance by showing that ageism is not experienced uniformly across the city, but intensifies in contexts of greater socioeconomic vulnerability, where it intersects with territorial disadvantage and reinforces processes of exclusion. In this sense, ageism was described not only in interpersonal terms, but also through everyday arrangements in services and the built environment that fail to accommodate older people’s needs, particularly in more vulnerable urban settings.

In addition to these transversal axes, a set of cross-cutting experiences further enriched the analysis by drawing attention to dimensions less frequently addressed in Latin American studies on urban ageing. These included emotional wellbeing linked to companionship—both human and non-human, such as relationships with pets—as well as experiences of loneliness and socioeconomic precariousness expressed through informal survival strategies. Education for later life also emerged as a relevant transversal process, understood as a holistic endeavour encompassing health, finances, social relationships, and digital literacy. Rather than constituting separate analytical domains, these experiences permeated multiple dimensions of everyday urban life, revealing how age-friendliness was lived through affective, relational, and symbolic processes alongside material conditions.

Nevertheless, despite its strength in providing an in-depth understanding of contrasting urban realities, the study has certain limitations. First, the territorial scope of the findings was restricted, limiting their transferability to other urban contexts. Second, the information reflected a specific moment in time, without allowing for longitudinal observation. Finally, although service providers participated, their voices were less represented compared to those of older people and caregivers, leaving gaps in the institutional perspective; therefore, interpretations related to institutional practices should be read as indicative of the experiences reported by this subgroup rather than as system-level conclusions.

Future research could expand geographical coverage to other Latin American cities, incorporating intersectional approaches (gender, disability, ethnic belonging) and mixed methods that allow for inter-urban comparisons and more comprehensive analyses of urban age-friendliness. It is also pertinent to evaluate the impact of specific interventions in mobility, healthcare, and digital literacy, as well as to deepen the qualitative understanding of service providers’ experiences, in order to capture everyday challenges more fully and generate evidence useful for guiding inclusive policies and programmes.

Finally, this study contributes to the field of urban age-friendliness by showing, from a qualitative perspective, how intra-urban territorial inequality and ageism operate as transversal axes that organise older people’s everyday experiences within the same city—an aspect still scarcely documented in Latin American literature. Rather than acting independently, these axes intersect to shape differentiated trajectories of mobility, participation, access to services, and recognition, producing ambivalent and uneven perceptions of age-friendliness across urban contexts.

By integrating dimensions such as ageism, mobility, health, participation, and access to information, it provides a broad understanding of the factors that shape wellbeing in later life. Taken together, the findings underscore that urban age-friendliness should be understood as a social and political process rather than a purely infrastructural endeavour. Recognising the ambivalent ways in which ageing is experienced—shaped by intra-urban territorial inequalities and ageism—is essential for informing public policies that are responsive to older people’s lived realities and capable of advancing integral, intersectoral, and territorially grounded approaches to ageing in the city.

## Conclusion

The findings of this study provide an exploratory and context-specific understanding of the complexity involved in addressing the needs of older people in a city such as Bogotá. Across the three localities analysed, intra-urban territorial inequalities and ageism shaped differentiated experiences of quality of life, in which inclusion and exclusion coexisted within and across localities.

Rather than operating independently, these processes interacted across everyday urban domains, influencing how older people moved through the city, participated in social life, accessed services, and were recognised in public and institutional spaces. This interaction helps explain why urban age-friendliness was experienced as uneven and, at times, contradictory within the same city.

In this context, the study draws attention to intra-urban diversity and to experiences of ageism, socioeconomic precariousness, and the digital divide, as well as to the ways in which older people’s voices are incorporated into urban life. Urban age-friendliness thus emerges not as a fixed condition, but as a social and political process shaped by everyday interactions, territorial contexts, and institutional arrangements.

Finally, building on the exploratory and context-specific nature of this study, future research could focus on comparative analyses across urban contexts, longitudinal qualitative approaches to capture changes over time, and mixed-methods designs that further examine the ambivalent dynamics of urban age-friendliness. Such approaches would allow a deeper understanding of how inequalities, institutional arrangements, and everyday experiences of ageing interact across different cities and policy settings.

## Data Availability

The transcripts and other materials generated and analysed during the study are not publicly available in order to safeguard participants’ confidentiality and privacy.

## Abbreviations

WHO: World Health Organization
PAHO: Pan American Health Organization
DANE: National Administrative Department of Statistics
EPS: Health Insurance Providers
JAC: Community Action Boards
COREQ: Consolidated Criteria for Reporting Qualitative Research
NVivo: NVivo qualitative data analysis software
